# Diazotrophic Macroalgal Associations With Living and Decomposing *Sargassum*

**DOI:** 10.3389/fmicb.2018.03127

**Published:** 2018-12-18

**Authors:** Yubin Raut, Michael Morando, Douglas G. Capone

**Affiliations:** Marine and Environmental Biology, University of Southern California, Los Angeles, CA, United States

**Keywords:** heterotrophic nitrogen fixation, diazotrophic macroalgal associations (DMAs), *Sargassum*, decomposition, sulfate reducers

## Abstract

Despite several studies reporting diazotrophic macroalgal associations (DMAs), biological nitrogen fixation (BNF) is still largely overlooked as a potential source of nitrogen (N) for macroalgal productivity. We investigated the role of BNF, via the acetylene reduction method, throughout different life stages of the invasive macroalga, *Sargassum horneri*, in its non-native Southern California coastal ecosystem. Throughout most of its life cycle, BNF rates were not detectable or yielded insignificant amounts of fixed N to support *S. horneri* productivity. However, during late summer when nutrient concentrations are usually at their minimum, BNF associated with juvenile *S. horneri* contributed ∼3–36% of its required N, potentially providing it with a competitive advantage. As DMAs remain poorly understood within macroalgal detrital systems, long term (15–28 days) laboratory decomposition time series were carried out to investigate the role of BNF throughout decomposition of the endemic macroalga, *S. palmeri*, and the invasive *S. horneri*. Nitrogenase activity increased drastically during the second phase of decomposition, when increasing microbial populations are typically thought to drive macroalgal degradation, with BNF rates associated with *S. palmeri* and *S. horneri* reaching up to 65 and 247 nmol N × g^-1^(dw) × h^-1^, respectively. Stimulation of BNF rates by glucose and mannitol additions, up to 42× higher rates observed with *S. palmeri*, suggest that labile carbon may be limiting at varying degrees in these detrital systems. Comparable, if not higher, dark BNF rates relative to light incubations during *S. horneri* decomposition suggest an important contribution from heterotrophic N fixers. Inhibition of nitrogenase activity, up to 98%, by sodium molybdate additions also suggest that sulfate reducers may be an important constituent of the detrital diazotrophic community. As labile N can become limiting to the microbial community during macroalgal decomposition, our results suggest that BNF may provide a source of new N, alleviating this limitation. Additionally, while BNF is rarely considered as a source for N enrichment with aging macroalgal detritus, we found it to account for ∼1–11% of N immobilized with decaying *S. horneri*. Our investigations suggest that DMAs may be globally important with *Sargassum* and potentially occur within other macroalgal detrital systems.

## Introduction

Benthic and pelagic marine macroalgae, encompassing three divisions (Rhodophyta, Chlorophyta, Phaeophyceae), are a diverse group of photoautotrophic organisms that extend across polar, temperate, and tropical latitudes (Keith et al., 2014). Species of brown macroalgae from the genus *Sargassum* truly highlight this cosmopolitan distribution as they occur in diverse benthic habitats along coastal zones throughout the world, spanning from the Gulf of California (Pacheco-Ruíz et al., 1998) to the rocky intertidal shores of the Japan Sea (Umezaki, 1984), occur in coral reefs such as the Great Barrier Reef (McCook, 1997) and form pelagic communities throughout the Sargasso Sea, Gulf of Mexico, and Gulf Stream (Butler and Stoner, 1984). Additionally, substrate-attached benthic macroalgae are often uprooted via different processes, e.g., exposure to waves and currents, storm events, or grazing, and transported offshore where they proliferate as rafts of drifting seaweed (Komatsu et al., 2008).

Benthic macroalgae can make important seasonal contributions to primary productivity in nearshore fringing reefs of the central Great Barrier Reef (Schaffelke and Klumpp, 1997) and dominate productivity along coastal ecosystems, contributing up to 50% of global coastal gross primary production (Duarte et al., 2005). Similarly, pelagic *Sargassum* communities can also make important contributions to upper ocean primary production with an input of ∼60% of total primary production in the upper 1m of the water column in the Sargasso Sea (Coston-Clements et al., 1991). Although primary production by macroalgae provides fixed carbon to other organisms through leaching of dissolved organic carbon or direct grazing, the vast majority of macroalgal production (estimates up to 90%; Mann, 1973) ultimately enters the detrital food chain or is sequestered in marine sediments or the deep ocean (Krause-Jensen and Duarte, 2016).

Macroalgae also create diverse habitats ranging from small drifting clumps of seaweed to structurally complex three dimensional communities such as giant kelp forests. These communities can host a rich assemblage of micro- and macro-organisms (e.g., prokaryotic and fungal epiphytes, a wide array of fishes, turtles and other vertebrates, gastropods, amphipods, nematodes, hydrozoans, and other invertebrates) that vary from larvae to juveniles to adults (Ingólfsson, 2000; Graham et al., 2007; Goecke et al., 2010; Abé et al., 2013). Furthermore, macroalgal habitats (e.g., *Sargassum* beds) can also control nutrient concentrations, pH, dissolved oxygen (O_2_) concentrations, and photon flux in the immediate ecosystem surrounding it (Komatsu, 1989; Lapointe, 1997; Zhang et al., 2008). The myriad of ecological functions that benthic, pelagic, or drifting macroalgae serve exemplifies their versatility and importance in marine ecosystems.

The success of benthic and pelagic macroalgae, such as the globally relevant *Sargassum* species, are largely dependent on the availability of light and nutrients. With regard to nutrients, nitrogen (N) is usually reported as a major controlling factor in the growth cycle of macroalgae and oftentimes, it is the dominant limiting factor for macroalgal productivity (Gagné et al., 1982; Hanisak, 1983; Fujita et al., 1989; Pedersen and Borum, 1996; Larned, 1998; Zhang et al., 2008). Macroalgae obtain N, along with other nutrients, from the water column where N can accumulate via different avenues including seasonal and local upwelling events (Gagné et al., 1982; Fujita et al., 1989), winter mixing events (Chapman and Craigie, 1977), nutrient excretions by associated organisms (Lapointe et al., 2014), nutrients from the underlying sediments, and eutrophication from N loading events such as wastewater discharge (Valiela et al., 1997).

Biological N fixation (BNF) is the conversion of dinitrogen gas (N_2_) into a biologically available form of N (e.g., NH_3_) mediated by a specialized group of prokaryotic organisms known as diazotrophs. Diazotrophs use the nitrogenase metalloenzyme to tap into the vast dissolved reservoir of N_2_ and provide a new source of N, making significant contributions to the global N budget and primary production (Capone, 2001). BNF associated with pelagic *Sargassum* species (i.e., *S. fluitans*, *S. natans*) has been examined by a number of investigators (e.g., Carpenter, 1972; Hanson, 1977; Phlips et al., 1986) with varying estimates of contributions of fixed N to sustain macroalgal productivity. In contrast, studies on benthic *Sargassum* species are less frequent (Phlips et al., 1986; Odintsov, 1992). Despite several additional accounts [as determined via the acetylene reduction (AR) method or stable isotope approach] of diazotrophic macroalgal associations (DMAs) observed with other benthic macroalgae (1) *Codium decorticatum* (Rosenberg and Paerl, 1981), (2) *C. fragile* (Head and Carpenter, 1975; Gerard et al., 1990), (3) *Microdictyon* sp., *Dictyota* sp. (Capone et al., 1977), (4) *Acanthophora* sp. (Goldner, 1980; France et al., 1998), (5) *Laurencia* sp. (Capone, 1977), and (6) *Caulerpa* sp. and *Lobophora* sp. (Tilstra et al., 2017), BNF is still rarely considered as a potential source of N for macroalgal productivity.

While there are a limited number of studies investigating BNF associated with living macroalgae, it remains virtually unexplored with decomposing macroalgal systems. In contrast, BNF dynamics have been routinely investigated with other macrophyte litter such as mangrove leaf detritus and senescing sea grass meadows which have repeatedly been shown to support diazotrophic activity (Gotto and Taylor, 1976; Zuberer and Silver, 1978; Capone and Budin, 1982; O’Donohue et al., 1991). The only prior publication (to our knowledge) that directly investigated BNF activity associated with decomposing seaweed reported higher BNF rates associated with *Macrocystis pyrifera* detritus compared to healthy *M. pyrifera* blades (Hamersley et al., 2015). Hamersley et al. (2015) proposed that while BNF did not contribute significantly to the N quota required during macroalgal production, it played an important role with *M. pyrifera* post-senescence by potentially improving the nutritional quality of the degrading macroalgal substrate as it entered the detrital food web.

Although *Sargassum* beds in native ecosystems serve important ecological functions and environmental services, the roles of various introduced *Sargassum* species (e.g., *S. muticum, S. horneri*) within non-native macroalgal systems suggest a potentially harmful ecological impact (Stæhr et al., 2000; Cruz-Trejo et al., 2015; Caselle et al., 2018). The introduction of an invasive macroalga, *S. horneri*, to the eastern Pacific (Miller et al., 2007) and its increasing range expansion throughout the Southern California ecosystem (Cruz-Trejo et al., 2015; Marks et al., 2015; Kaplanis et al., 2016) provided us with a unique opportunity to tackle many of the aforementioned gaps in the literature regarding DMAs. Primarily, we sought to investigate the role of diazotrophs throughout the life cycle of *S. horneri* to assess whether BNF provided an advantage to this globally important macroalga in a non-native ecosystem, thereby also expanding upon the limited literature regarding BNF associated with benthic *Sargassum* species in general. As BNF dynamics associated with decomposing macroalgal systems remain poorly understood, we also thought it pertinent to (1) investigate BNF associated with aging *S. horneri* detritus in comparison to other endemic species in the Southern California coastal ecosystem (e.g., *S. palmeri*) and (2) explore if diazotrophs may influence the nutritional quality of *S. horneri* detritus.

## Materials and Methods

### Sample Collection and Experimental Set Up

SubstrateWe have changed Methods as Materials and Methods. Kindly confirm if this is fine. attached *S. horneri* at various life stages, i.e., juvenile, immature, mature, and senescent, were collected throughout different seasons over 2 years (2016 and 2017) at various locations surrounding Santa Catalina Island, CA, United States (Supplementary Table S1). Substrate attached *S. palmeri* that was beginning to senesce was also collected during the summer of 2016 (Supplementary Table S1). Upon collection, most samples were transported back to the Wrigley Marine Science Center where the bulk of the experiments and incubations took place. However, there were some instances when fresh samples were transported back to the University of Southern California (USC) main campus in a bucket of seawater that was aerated with a bubbling stone prior to experimental setup.

For long term laboratory decomposition experiments, freshly collected samples were randomly separated into multiple 200 μm white mesh litter bags and allowed to degrade inside continuous flow-thru seawater tanks. The 2016 experiments took place in outdoor tanks with shade screening resulting in ∼40–60% of ambient surface photosynthetically active radiation (PAR), resembling conditions experienced by floating rafts of *S. horneri*. The 2017 experiments took place in indoor tanks without any screening resulting in ∼4–10% of ambient surface PAR, resembling decomposition conditions more likely to exist in the benthos. A different litter bag was sub-sampled every few days and the macroalgal contents were apportioned into homogenous groups that were then incubated for ∼24–48 h in serum vials with different amendments for quantification of BNF rates via the AR method (discussed below). In order to track loss of biomass, the same litter bag was manually squeezed to remove excess water and the wet weight was recorded every few days over a 20-day period. The initial weight is considered to be at full saturation and every weight measured thereafter is reported as the percentage of biomass remaining relative to the initial weight (Supplementary Figure S1).

In the 2016 decomposition experiment, *S. horneri* and *S. palmeri* were sub-sampled for AR assays on days 0, 5, 11, 21, and 28. All serum vials used for AR assays were incubated in the same outdoor tanks where macroalgal litter bags were undergoing long term decomposition. In the 2017 decomposition experiment, *S. horneri* was sub-sampled on days 0, 3, 8, 12, and 15 for AR assays. These serum vials were incubated inside a temperature and light controlled incubator held at a constant 18°C with 12-h diel cycles at ∼3–10% of ambient surface PAR. From seasonal field sampling efforts, fresh macroalgal samples were placed immediately (∼2–5 h upon collection) in serum vials and assayed for nitrogenase activity to quantify ambient BNF rates. Similar to the decomposition experiment, these serum vials underwent short-term incubations for ∼24–48 h either in the flow-thru seawater tank inside the laboratory (∼4–10% PAR) or in a 16°C temperature controlled incubator with 12-h diel cycles at ∼5% PAR.

### Light vs. Dark Assays

Parallel light and dark assays took place for all fresh and long-term decomposition experiments. All light assays were set up by placing macroalgal samples in clear 14 or 27 mL serum vials with 0.2 μm filtered seawater without any further changes to the illuminance. Dark assays took place in 14 or 27 mL serum vials wrapped in aluminum foil with 0.2 μm filtered seawater. For the 2016 decomposition experiment of *S. horneri* and *S. palmeri*, dark vials were also purged with N_2_ gas for ∼1.5 min before beginning the AR assays. Dark vials for the 2017 *S. horneri* decomposition experiment were not purged with N_2_ gas and light inhibition remained to be the only perturbation to these assays.

### Carbon Additions: Glucose and Mannitol Amendments

Primary stocks of D-glucose (C_6_H_12_O_6_) and mannitol (C_6_H_14_O_6_) were made in the same batch of 0.2 μm filtered seawater as what was used for incubating macroalgae in the serum vials for the AR assays. For the 2016 *S. horneri* and *S. palmeri* decomposition experiments, glucose and mannitol amendments at different concentrations were carried out in in both light and dark vials in parallel to the control light and dark assays. On day 0, 100 μM of glucose and mannitol was added to all assays. However, glucose and mannitol amendments were increased to 1mM additions for all subsequent assays (i.e., days 5, 11, 21) throughout the decomposition experiment except on day 28 when only control assays were carried out due to a shortage in remaining biomass to carry out the carbon amendments.

### Sodium Molybdate Assays

Primary stocks of sodium molybdate dihydrate (Na_2_MoO_4_⋅2H_2_O), a known inhibitor of sulfate reducing bacteria (Oremland and Capone, 1988), were made in the same batch of 0.2 μm filtered seawater as what was used for incubating macroalgae in the serum vials. For the duration of the 2017 *S. horneri* decomposition experiment (i.e., days 0, 3, 8, 12, and 15), 20 mM sodium molybdate amendments were carried out in both light and dark vials concurrent to control light and dark assays.

### AR Assay

The widely used AR assay was utilized to quantify ethylene (C_2_H_4_) production and assess nitrogenase activity (Capone, 1993). For each amendment (e.g., control light/dark, glucose light/dark, etc.), triplicate 14 or 27 mL serum vials were capped with gray butyl stoppers and crimped with aluminum crimp caps before injecting with 1 or 2 mL of acetylene (C_2_H_2_) gas (produced by reacting water with calcium carbide) using a disposable BD syringe with luer lock tips. Regardless of the size of the serum vial (14 vs. 27 mL), the ratio of aqueous to gas phase was kept constant at ∼67:33%, respectively, and the volume of C_2_H_2_ injected (∼20% of the gas phase or ∼7% of total volume) was enough to saturate the nitrogenase enzyme (Flett et al., 1976). The vials were gently shaken (∼5 inversions) upon injection of C_2_H_2_ to equilibrate the vapor phase with the aqueous phase and incubated for ∼24–48 h in the aforementioned temperature and light conditions. Negative controls without any C_2_H_2_ introduced into the serum vials containing macroalgae were also routinely tested in order to ensure there was no background C_2_H_4_ production during these incubations.

Starting with a T_0_ time-point upon initial C_2_H_2_ injection, 100 μL subsamples were taken from the headspace of all replicates using a Hamilton gas tight syringe and injected into a gas chromatograph (Shimadzu Mini 2) equipped with a flame ionization detector at different time-points (every ∼3–8 h) throughout the duration of the incubation. The PeakSimple chromatography data system was used to quantify C_2_H_4_ peak heights at these different time-points and subsequently converted to nmol of C_2_H_4_ produced (Capone, 1993; Breitbarth et al., 2004). Instantaneous rates between time-points which contributed less than 30% to the overall rate were used to identify occurrences of severe lags in the beginning of incubations and plateaus toward the end of an incubation. Finally, rates of C_2_H_4_ production excluding lags and plateaus were determined using linear regressions and subsequently, theoretical BNF rates were calculated using a 3:1 C_2_H_2_ reduced:N_2_ reduced ratio (Capone, 1993) and multiplied by 2 to express as nmol N × g^-1^(dry weight, dw) × h^-1^.

### C and N Content and δ^15^N Analysis

Throughout the 2017 *S. horneri* decomposition experiment, fresh detritus (FD) was subsampled on days 0, 3, 8, 12, and 15 for CN and δ^15^N isotope analysis. Additionally, a portion of the FD subsampled on the aforementioned days were incubated for ∼48 h for AR assays and retrieved on days 2, 5, 10, 14, and 17 for CN analysis. These post-incubation detritus samples will be referred to as PID. Dried macroalgal samples, stored in aluminum pockets were transported back to the USC main campus where they were homogenized to a fine powder using a mortar and pestle. All samples ranging between ∼1 and 1.3 mg were then encapsulated in tin capsules and pelletized for elemental (CN) and/or isotopic (δ^15^N) analysis on a Micromass IsoPrime continuous flow isotope ratio mass spectrometer with CHN analyzer/sample front ends.

### Light and O_2_ Measurements

PAR for the surface ocean (<1 ft.), outdoor tank, indoor tank, and incubator was measured at the same time (within 10 min) using a handheld quantum PAR meter (Biospherical Instruments Inc., San Diego, CA, United States) over multiple days. The surface ocean readings were assumed to be fully saturated and all other conditions are expressed as a percentage relative to the ambient surface conditions from that day. Dissolved O_2_ concentrations inside an open 27 mL serum vial with 0.2 μm filtered seawater and senescent *S. horneri* or without any *S. horneri* were measured using an O_2_ microelectrode (Ox-50, Unisense A/S, Aarhus, Denmark). Multiple depth profiles, over ∼30 mm, were taken at 1 mm intervals and several depth profiles at close proximity to the seaweed were also taken at the μm scale using the micromanipulator (Unisense A/S).

### Statistical Analysis

Since not all assumptions (i.e., normality, homoscedasticity) were met for standard tests such as ANOVA, non-parametric equivalents of the one-way ANOVA (Wilcox, 2012b) and two-way ANOVA (Wilcox, 2012c) were utilized to analyze the data sets for BNF rates, %C, %N, and C:N ratios. In conjunction with these analyses, a non-parametric equivalent to the classic Tukey’s HSD *post hoc* analysis which allows heteroscedasticity, lincon function on the R software (Wilcox, 2012a), was utilized to conduct pairwise comparisons between various treatments for the different data sets. Statistically significant differences for *p*-values less than 0.05, 0.01, and 0.001 are included in the various figures.

## Results

### Role of BNF During Different Life Stages of *S. horneri*

**FIGURE 1:**
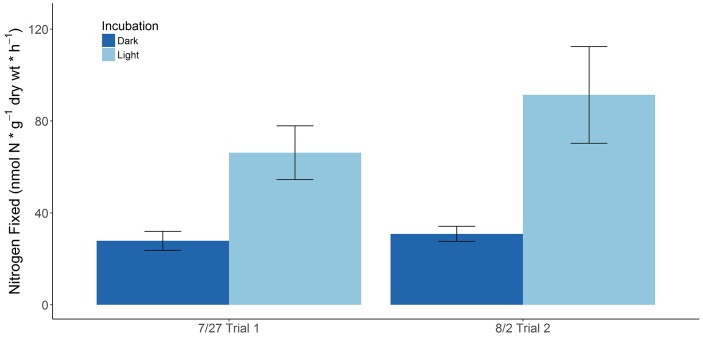
BNF rates associated with juvenile *S. horneri* recruits on two separate trials (error bars represent SE).

Seasonal BNF rates associated with freshly collected *S. horneri* (Supplementary Table S2) yielded significantly lower rates than those associated with decomposing macroalgal detritus (Supplementary Tables S3, S4). BNF rates were not detectable with juvenile and immature *S. horneri* collected during the fall and winter. However, BNF rates close to the limit of detection [∼0.7–2 nmol N × g^-1^(dw) × h^-1^] were observed with adult *S. horneri* toward the end of winter. Higher BNF rates [∼12–24 nmol N × g^-1^ (dw) × h^-1^] associated with freshly collected *S. horneri* were measured in the summer when necrosis was already beginning to take place with the seaweed upon time of collection. The highest BNF rates [∼28–91 nmol N × g^-1^(dw) × h^-1^] with living *S. horneri* was associated with juveniles in the late summer (Figure 1).

Relative growth rates expressed in terms of percent increases in blade weight or fresh weight per day, ranging from 4 to 5.2% (Gao and Hua, 1997; Choi et al., 2008), were used to estimate increases in biomass of whole juveniles collected in this study as g (dw) per day. These growth rates were found to be in the same range as growth rates of different *S. horneri* juveniles reported in other studies (Umezaki, 1984; Yoshida et al., 2001), ranging from 0.003 to 0.01 g (dw) per day (Supplementary Table S5). In concert with the estimated and previously reported growth rates of *S. horneri* juveniles from approximately the same time, the average %N (∼1.01 ± 0.04%, 1.07 ± 0.07%) of *S. horneri* juveniles collected in July and August, respectively, were used to calculate g of N required to sustain growth of *S. horneri* juveniles. As both trials in July and August yielded higher BNF rates in the light than the dark, the average dark and light BNF rate [∼47 and 61 nmol N × g^-1^ (dw) × h^-1^, respectively] was assumed to represent the overall diazotrophic activity in a full day. This was used to calculate potential contributions of fixed N from diazotrophic activity to meet N demands for *S. horneri* juveniles during this period. The contribution of fixed N from diazotrophic activity ranged from 3 to 36% with a mean contribution of ∼9.6%. The results from this analysis can be found in the Supplementary Table S6.

**FIGURE 2:**
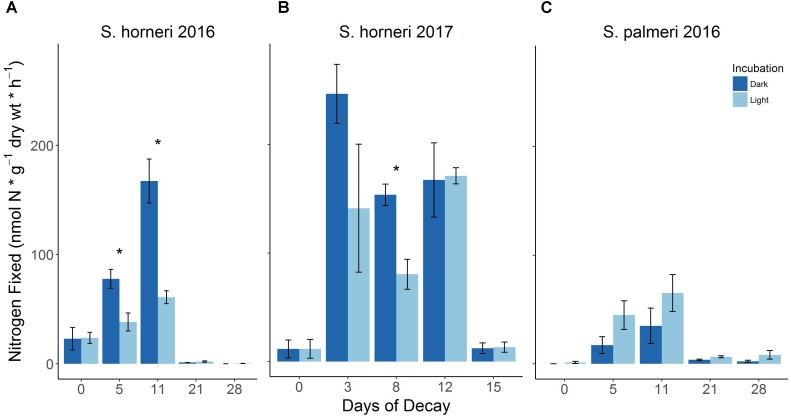
BNF rates associated with decomposing *S. horneri* **(A,B)** and *S. palmeri* **(C)** under dark and light incubations during two summers (2016 and 2017) (error bars represent SE). ^∗^*p* < 0.05.

### Trends in Diazotrophic Activity Throughout Macroalgal Decay

Two separate laboratory decomposition experiments carried out in subsequent summers showed recurring shifts in diazotrophic activity associated with the degradation of the invasive macroalga, *S. horneri* (Figures 2A,B). Initially, when seaweed samples were relatively fresh and only beginning to senesce, lower BNF rates were observed [∼12–24 nmol N × g^-1^(dw) × h^-1^]. Following just a few days (∼3–5 days) of decomposition during which there was ∼20% loss in biomass (Supplementary Figure S1), there was a distinct increase (up to 10×) in diazotrophic activity with BNF rates reaching up to 247 nmol N × g^-1^(dw) × h^-1^ (Figures 2A,B). This increase in nitrogenase activity was sustained for a number of days (until day ∼11–12) by which time there was an additional ∼40% loss in biomass (Supplementary Figure S1). This trend in rapid biomass loss continued from day 12 to 14 during which time there was a further ∼9% loss in biomass (Supplementary Figure S1). The ensuing plateau in macroalgal degradation from days 14 to 20, with only ∼1% loss in biomass, coincided with a substantial decrease in diazotrophic activity (∼day 15), resulting in BNF rates comparable to the initial rates or in some cases, lower than the initial rates (Figures 2A,B and Supplementary Figure S1). These same trends in diazotrophic activity were also observed during decomposition of the endemic *Sargassum* species, *S. palmeri* (Figure 2C). Lastly, while several instances of *S. horneri* dark assays yielded higher BNF rates, dark assays of *S. palmeri* did not result in higher nitrogenase activity than light assays (Figure 1). All calculated BNF rates for control treatments are reported in the Supplementary Tables S3, S4.

**FIGURE 3:**
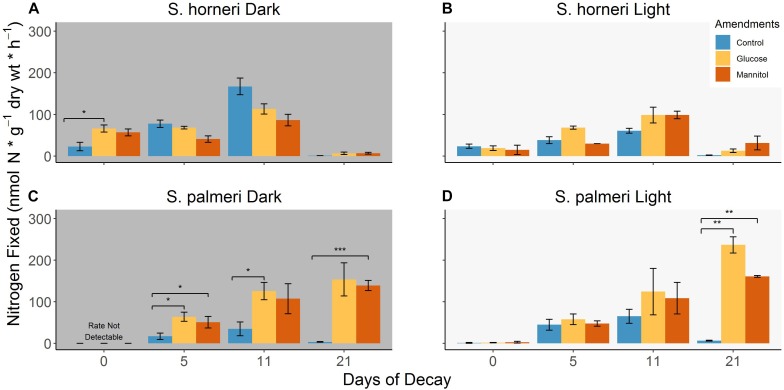
**(A–D)** Differences in BNF rates associated with decomposing *S. horneri* and *S. palmeri* between control conditions, glucose (100 μM/1 mM) and mannitol (100 μM/1 mM) additions under dark and light incubations (error bars represent SE). ^∗^*p* < 0.05, ^∗∗^*p* < 0.01, ^∗∗∗^*p* < 0.001.

### Impact of Carbon Additions on BNF Rates

For *S. horneri*, we observed that additions of 100 μM glucose and mannitol on day 0 stimulated nitrogenase activity in the dark assays by a factor of ∼3 and 2.5, respectively, but no stimulation of BNF rates were observed in the light assays (Figures 3A,B). In contrast, there were low to non-detectable BNF rates associated with *S. palmeri* with the same carbon amendments (Figures 3C,D). Higher concentrations of glucose (ranging from ∼56 μM to 56 mM) have been used in prior investigations looking at DMAs (Head and Carpenter, 1975) and thus, in order to ensure an increase in measurable nitrogenase activity, the amendment concentration for both glucose and mannitol were increased to 1 mM for all subsequent days which yielded mixed results.

In both light and dark assays for *S. horneri*, adding 10× more glucose and mannitol failed to further stimulate the diazotrophic community associated with it until day 21. On day 21, glucose and mannitol amendments in the dark and light assays stimulated BNF rates by 5.3–15, but even these elevated rates were either comparable to or lower than the control rates observed earlier in the decomposition period (Figure 3A). For *S. palmeri*, higher concentrations of glucose and mannitol stimulated BNF rates (∼3.7- and 3-fold, respectively) on day 5 in dark assays but failed to further stimulate nitrogenase activity in the light assays (Figures 3C,D). However, for the remainder of the decomposition period of *S. palmeri*, carbon amendments greatly stimulated BNF rates (with up to ∼42-fold stimulation) in both light and dark assays (Figures 3C,D). A complete list of BNF rates for various amendments with both species of macroalgae are available in the Supplementary Table S3.

**FIGURE 4:**
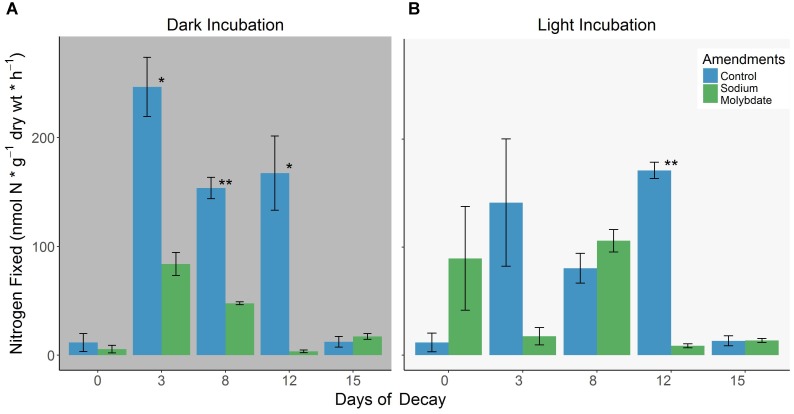
**(A,B)** Differences in BNF rates associated with decomposing *S. horneri* between control conditions and sodium molybdate (20 mM) additions under dark (left) and light (right) incubations (error bars represent SE). ^∗^*p* < 0.05, ^∗∗^*p* < 0.01.

### Impact of Sodium Molybdate Amendments on Nitrogenase Activity

It is evident in the dark assays that the addition of 20 mM sodium molybdate routinely suppressed nitrogenase activity throughout most of the decomposition period and with increasing intensity, reaching the highest reduction (∼98%) in BNF rates on day 12 (Figure 4A). The same amendments produced mixed results in the light assays but apart from day 0 where there was stimulation of nitrogenase activity, it either severely inhibited (maximum reduction of ∼95% on day 12) or did not significantly impact nitrogenase activity (Figure 4B). The complete list of BNF rates are available in the Supplementary Table S4.

**FIGURE 5:**
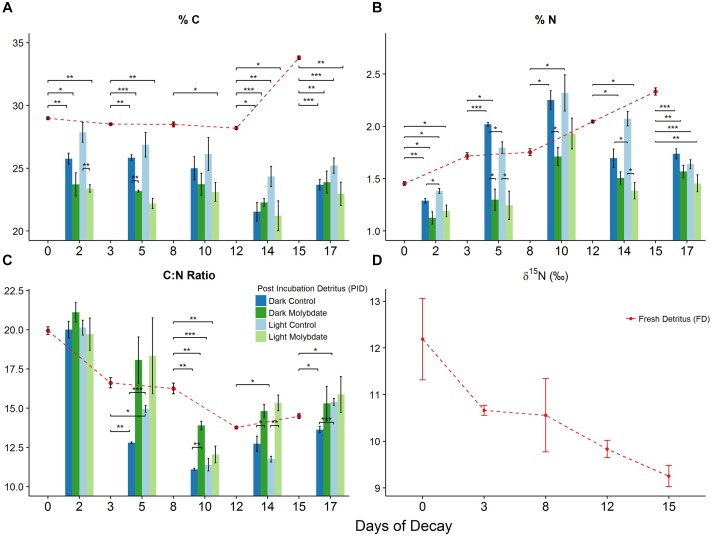
Changes in %C **(A)**, %N **(B)** and C:N ratio **(C)** associated with decomposing *S. horneri* in control (blue) and molybdate amendments (green) in both dark and light incubations as well as pre-incubation samples (red dashed line) (2017). Additional changes in δ^15^N **(D)** with fresh detritus throughout decomposition (error bars represent SE). ^∗^*p* < 0.05, ^∗∗^*p* < 0.01, ^∗∗∗^*p* < 0.001.

### Changes in C and N Content (%), C:N Ratio and δ^15^N Signature of Aging *S. horneri* Detritus

There is an overall decrease in C, by 2.73%, with fresh *S. horneri* detritus subsampled over the first 12 days of decomposition (Figure 5A). In contrast, N increases by 40.8% with FD during this time (Figure 5B). Between day 12 and 15, FD exhibited a sudden increase in C by 20% and N continued to increase by 14% (Figures 5A,B). The resulting C:N ratio with FD decreased by 31% over the first 12 days of decomposition and despite increasing from day 12 to 15 by 5.2%, the final C:N ratio exhibited an overall decrease of 27.3% (Figure 5C). The δ^15^N of FD continuously decreased from 12.2‰ to 9.3‰ over the course of the decomposition, resulting in an overall decrease of 24.1% by day 15 (Figure 5D).

There is also an overall decrease in C, between 6.1 and 16.4%, with PID recovered at the end of ∼48-h dark and light incubations under control and sodium molybdate treatments over the first 12 days of decomposition (Figure 5A). Similar to the increase observed with day 15 FD, PID recovered at the end of this incubation also exhibited an increase in C, between 3.5 and 10%, amongst all treatments (Figure 5A). All PID exhibited increases in N, between 52.2 and 74.7%, over the first 8 days of decomposition (Figure 5B). Subsequently, there was an overall decrease in N, between 8.4 and 29.4%, with all PID over the remainder of the decomposition experiment (Figure 5B). Despite this decrease at the end, all PID were still relatively enriched, by 18.5 to 39.4%, in N compared to PID from the onset of decomposition (Figure 5B). The resulting C:N ratio for all PID exhibit an inverse pattern to the %N trends, expressing an overall decline over the first 8 days of decomposition followed by an increase until the end of the decomposition experiment (Figure 5C). Despite this increase at the end, there is an overall decrease with the C:N ratio of PID at the end of decomposition relative to the initial (day 2) PID (Figure 5C). The PID recovered from sodium molybdate treatments resulted in higher C:N ratio compared to the control treatments throughout the duration of the experiment (Figure 5C).

Control and sodium molybdate incubations for ∼48 h in the dark and light repeatedly resulted in PID that was more depleted, between 3.9 and 32.1%, in C relative to the FD (Figure 5A). For the most part, sodium molybdate incubations in the dark and light also resulted in PID that were more depleted, between 5.1 and 17.4%, in C compared to control PID from parallel incubations (Figure 5A). While control PID in the light repeatedly resulted in higher %C than the dark incubations, the opposite trend was observed with sodium molybdate treatments which consistently resulted in slightly higher %C with substrate recovered from dark incubations (Figure 5A).

Control incubations in the dark and light resulted in PID that were depleted, between 4.8 and 29.7%, in N compared to FD at the beginning (day 0) and end (day 15) of decomposition (Figure 5B). However, PID retrieved at the end of day 3 and day 8 from dark and light control incubations exhibited higher N, between 4.6 and 32.4%, relative to the FD collected on those corresponding days (Figure 5B). While PID recovered at the end of day 12 light incubations resulted in slightly higher N, by 1.26%, PID from dark incubations were 17.1% more depleted in N compared to the FD collected initially on day 12 (Figure 5B). Apart from day 8 when PID in the light were more enriched in N, by 10.1%, PID recovered at the end of both light and dark sodium molybdate incubations were more depleted in N, between 2.4 and 37.6%, compared to corresponding FD subsampled on days 0, 3, 8, 12, and 15 (Figure 5B). For both dark and light incubations, PID from sodium molybdate treatments always resulted in lower N, between 9.8 and 35.7%, compared to control PID from parallel incubations (Figure 5B). The %C, %N, C:N ratio, and δ^15^N values for the aforementioned samples can be found in the Supplementary Table S7.

From all treatments throughout the decomposition experiment, there were several instances, on days 3 and 8, when PID exhibited higher mean %N relative to FD collected at the beginning of the incubation (Figure 5B). Assuming a dry weight of 1 g of *S. horneri* detritus, the theoretical mass of N (mg) required to result in the observed increase in %N at the end of the incubation was calculated. The average BNF rates associated with the corresponding treatments, expressed as nmol N × g^-1^(dw) × h^-1^, were transformed into nmol of N fixed by multiplying the BNF rates by the assumed dry weight of the *S. horneri* detritus (1 g) and length of the incubation (∼48 h) and subsequently converted to mg of N. Lastly, the percent contribution of BNF to N immobilization was calculated by dividing the theoretical mass of N (mg) required by the theoretical mass of N (mg) fixed during the incubation. The percent contribution to N immobilization by BNF ranged from 0.94 to 10.6% with an average contribution of 4.5%. The complete results can be found in the Supplementary Table S8.

## Discussion

### Role of BNF in N Acquisition by Living *S. horneri*

Throughout much of the life cycle of *S. horneri*, nitrogenase activity was not detectable or exhibited relatively low BNF rates that yielded insignificant amounts of fixed N to support macroalgal productivity. However, on two separate occasions, active BNF was observed with living *S. horneri* juveniles (5–8 cm long) collected during late July and early August of 2017 (Figure 1). Ambient nutrient concentrations are usually at their minimum during late summer in the Southern California Bight and one study conducted at Santa Catalina Island (same site as this study) found the low nutrient concentrations in the surrounding waters during this time to be insufficient to support maximal growth of the endemic macroalgae, *M. pyrifera* (Zimmerman and Kremer, 1984). Similarly, it may also be possible that the low nutrient conditions are not ideal to support growth of *S. horneri* juveniles. Thus, appreciable amounts of fixed N, ∼3–36% of required N with an average contribution of ∼9.6%, derived from diazotrophic activity during this time might potentially supplement their N requirement and thereby provide a competitive edge. These values are comparable to previous estimates of BNF contributions to sustain macroalgal productivity of various species such as *C. decorticatum* (∼5%; Rosenberg and Paerl, 1981), *Laurencia* sp. (14–18%; Capone, 1977), and pelagic *Sargassum* community (∼40%; Hanson, 1977). Subsequent sampling efforts of juveniles in mid-September, October and late-November resulted in undetectable rates of BNF. Substantial increases in nutrient concentrations during the fall and winter, with maximum nitrate concentrations observed during this time (Zimmerman and Kremer, 1984) may explain the absence of BNF activity associated with *S. horneri* and potential shift back to nutrient uptake from the surrounding water column to sustain its growth and productivity.

### Significance of BNF Activity Associated With Decomposing *Sargassum* Detritus

It is well established that various marine macrophytes such as salt marsh grasses (Valiela et al., 1985), mangrove leaves (Cundell et al., 1979) and sea grasses (Godshalk and Wetzel, 1978) undergo an initial phase of rapid biomass loss due to autolysis and leaching of soluble compounds. This is followed by a longer phase of biomass loss driven by microbial degradation of organic matter before entering the slowest phase of decay due to the refractory nature of the remaining detritus. Similarly, numerous studies have also observed macroalgal decay to follow this three-phase decomposition model (Hunter, 1976; Robinson et al., 1982; Smith and Foreman, 1984; Williams, 1984; Rieper-Kirchner, 1989). Furthermore, the trend of biomass loss observed in this study (Supplementary Figure S1) also adheres to this three-phase decomposition model.

Observations of microbial densities throughout these three phases have generally found there to be relatively low microbial biomass during the initial phase of decomposition. This is followed by a rapid increase in microbial colonization, indicative of a transition to microbial degradation of macroalgal detritus, with maximal microbial biomass being sustained for several days before microbial populations decrease, marking the final shift toward a more recalcitrant macroalgal detritus (Robinson et al., 1982; Rice and Hanson, 1984; Rieper-Kirchner, 1989; Sathe-Pathak et al., 1993). Recurring shifts in nitrogenase activity during decomposition of *S. horneri* and *S. palmeri* resemble this characteristic shift in microbial biomass, though with a much steeper decrease in BNF rates toward the end of the decomposition period (Figure 2). If the diazotrophic population is assumed to be changing in a similar fashion to the whole microbial community, the lower BNF activity observed at the beginning and end of decomposition could be attributed to potentially lower diazotrophic biomass present during the initial and terminal phases of decomposition. The BNF hot spot observed in the middle of decomposition may be a result of greater diazotrophic recruitment concomitant with increasing microbial populations that generally transpires during the second phase of macroalgal decomposition.

As most macroalgae have a relatively high C:N ratio (>20:1 as observed with *S. horneri* samples in this study), increased microbial colonization during macroalgal degradation and rapid utilization of resources would lead to depletion in N before C. This may result in N limitation of the organic rich macroalgal substrate, thereby creating a niche for N fixing organisms. Several studies have shown additions of N to support greater microbial populations associated with macroalgal detritus. Consequently, increased microbial biomass were observed to utilize more of the available detrital carbon, expediting macroalgal degradation and highlighting the potential for N limitation to retard microbial degradation and impact the overall rate of macroalgal decomposition (Tenore et al., 1979; Robinson et al., 1982; Rice and Hanson, 1984). Additionally, it is also well established that N enrichment observed with aging macroalgal detritus does not necessarily indicate an increasing pool of labile N (e.g., proteins, amino acids) but rather, it might be an accumulation of recalcitrant N which also results in N limitation (Rice, 1982). BNF may provide new, bioavailable N for the associated microbial community, possibly alleviating N limitation and stimulating microbial degradation of macroalgae.

Interestingly, BNF rates associated with freshly collected *S. horneri*, which has been noted to begin senescing in its non-native eastern Pacific habitat during the spring (Miller et al., 2007; Cruz-Trejo et al., 2015), expressed a seasonal increase toward the beginning of summer when it was physically observed to be senescent. Similarly senescing *S. horneri* in the western Pacific (Yoshida et al., 1998, 2001) might also potentially support diazotrophic activity as they form drifting rafts during spring to early summer (Komatsu et al., 2007, 2008; Yatsuya, 2008; Mizuno et al., 2014). Additionally, despite having a relatively longer floating period due to its lower density (Yatsuya, 2008), *S. horneri* debris have also been found on the offshore deep sea floor during the summer (Kokubu et al., 2012). Therefore, it may also be possible that sinking *S. horneri* debris may further serve as a niche for diazotrophs in the deep sea benthos.

### Presence of Heterotrophic N Fixers

In different marine ecosystems such as the anoxic waters (200 m) of the Baltic Sea (Farnelid et al., 2013), aphotic zone (500 and 885 m) in the San Pedro Basin (Hamersley et al., 2011), and a wide assortment of benthic systems (Howarth et al., 1988), dark incubations have been utilized to estimate rates of heterotrophic BNF. While dark BNF rates could also include contributions from chemoautotrophic and methanotrophic diazotrophs, we are assuming BNF in the absence of light to be primarily a result of heterotrophic BNF. In this study, dark vials were purged with N_2_ gas in the 2016 *S. horneri* and *S. palmeri* decomposition experiment to promote an anaerobic environment and to protect the nitrogenase enzyme from oxygen toxicity and ensure measurable rates of BNF. While it could be argued that purging dark vials with N_2_ gas and leaving the light vials unperturbed might have resulted in elevated dark BNF rates in the first experiment, the 2017 *S. horneri* decomposition experiment, which utilized unpurged dark and light vials, also consistently exhibited higher rates of BNF associated with aging *S. horneri* detritus in the dark than in the light (Figures 2A,B). The second experiment, despite using unpurged vials, also expressed higher overall dark BNF rates compared to the 2016 experiment. Furthermore, similarly prepared dark, anaerobic assays routinely yielded lower BNF rates throughout the decomposition of *S. palmeri* (Figure 2C). These findings suggest that laboratory perturbations of dark vials in the first *S. horneri* experiment most likely did not provide any significant advantage to specific physiological groups of N fixers in the dark (Figure 2).

O_2_ depth profiles in an uncapped, illuminated serum vial containing senescent *S. horneri* samples in 0.2 μm filtered seawater showed that O_2_ concentrations drop well below hypoxic levels, <63 μM as defined for coastal ecosystems (Middelburg and Levin, 2009), when approaching the thicket of *S. horneri* but remain constant when only exposed to seawater (Supplementary Figures S2A,B). Furthermore, depth profiles measured using an O_2_ microelectrode reveal that O_2_ concentrations can drop close to 0 μM when in close proximity to senescent *S. horneri* particles (Supplementary Figures S2C,D). As observed with colonies of *Trichodesmium* (Paerl et al., 1989), localized areas of O_2_ consumption probably lead to formation of microaerobic niches with aging macroalgal detritus and provide protection from oxygen toxicity to diazotrophs. Additionally, heterotrophic respiration and inhibition of overall photosynthetic activity in the dark assays could have also prevented accumulation of high concentrations of O_2_ in the vials and further alleviated the nitrogenase enzyme from oxygen toxicity, resulting in higher rates of BNF in dark assays throughout the decomposition of *S. horneri*. As the exact nature of the diazotrophic population is currently unknown, N fixers in these detrital systems are most likely employing an array of methods to protect the nitrogenase enzyme from oxygen toxicity (Fay, 1992).

Similar to *S. palmeri*, dark BNF rates associated with living *S. horneri* juveniles were also consistently lower than in the light, suggesting a lesser contribution from heterotrophic N fixers in these systems (Figures 1, 2C). One possible explanation for higher dark BNF rates observed with decomposing *S. horneri* detritus compared to aging *S. palmeri* detritus might be differences in the diversity, abundance, and activity of the heterotrophic diazotrophs associated with the different macroalgae. Similarly, the difference in dark BNF rates associated with *S. horneri* at different life stages, i.e., juvenile vs. decomposing, leads us to hypothesize that there are most likely changes in the diazotrophic community composition associated with the host macroalga throughout its life cycle. This has been suggested with changes in the general microbial community associated with different macroalgae (Goecke et al., 2010) and future molecular work specifically targeting the *nifH* gene would better elucidate this hypothesis.

In many marine systems such as the oligotrophic open ocean and with previously reported DMAs (e.g., pelagic *Sargassum* species), phototrophic diazotrophs (e.g., *Trichodesmium, Crocosphaera, Calothrix, Dichothrix fucicola*) are often regarded as the most important contributors to BNF in the upper ocean (Carpenter, 1972; Phlips et al., 1986; Capone et al., 1997; Montoya et al., 2004). However, it seems heterotrophic diazotrophs may have an important association with decomposing *Sargassum* and *Macrocystis* (Hamersley et al., 2015), but with varying degrees of contribution to BNF. The possibility for DMAs to occur within numerous macroalgal detrital systems, as discussed previously, may provide an unexplored niche for heterotrophic N fixers, requiring additional investigation.

### Impact of Labile Carbon on Nitrogenase Activity

The concentrations of various organic molecules (e.g., alginates, phenols, total carbohydrates, proteins, reducing sugars, etc.) generally decline throughout macrophyte decomposition, resembling trends in loss of biomass throughout the three stages of decomposition (Cundell et al., 1979; Buchsbaum et al., 1991; Sathe-Pathak et al., 1993). Generally, rapid build-up in microbial biomass, observed after leaching of organic compounds during the first phase of decomposition, begins to decline as the detritus enters a more refractory phase, suggesting that the utilization of labile carbon is an important controlling factor for sustaining microbial populations during macrophyte decomposition (Cundell et al., 1979; Sathe-Pathak et al., 1993). Considering the high energetic cost of BNF and assuming the diazotrophic population is following similar changes in biomass to the whole microbial community, it is likely that the availability of various labile organic compounds may also have influenced nitrogenase activity associated with aging *S. horneri* and *S. palmeri* detritus.

During the first phase of *S. horneri* decomposition (∼days 0–5), the availability of labile carbon may have been limiting BNF in the dark, and thus, even relatively lower (100 μM) concentrations of glucose and mannitol were able to stimulate BNF on day 0 (Figure 3A). However, 10x higher (1 mM) glucose and mannitol additions did not significantly stimulate BNF throughout the second phase of decomposition (∼days 5–14), suggesting that availability of labile organic compounds during this phase were sufficient to support diazotrophic activity (Figures 3A,B). Interestingly, these same amendments in both the light and dark were able to stimulate diazotrophic activity, by ∼5- to 15-fold, associated with the more recalcitrant *S. horneri* detritus remaining on day 21 (Figures 3A,B). While this suggests that labile carbon may also be limiting BNF at the end of decomposition, these elevated BNF rates stimulated by carbon additions were relatively lower than control BNF rates observed throughout the second phase of decomposition (i.e., days 5, 11). This indicates a potential decrease in diazotrophic population and activity by the end of decomposition. In contrast, carbon additions routinely stimulated BNF rates, particularly in the dark, throughout the different stages of *S. palmeri* decomposition (Figures 3C,D). This suggests that the release of organic compounds throughout *S. palmeri* decomposition was not as accessible to the diazotrophic community, resulting in labile carbon limitation for N fixers associated with the aging *S. palmeri* detritus.

### Sulfate Reducing Diazotrophs

While there have been several studies reporting sulfate reduction associated with sediments in different macrophyte systems such as salt marshes (Hines et al., 1989), seagrasses (Capone, 1982; Blaabjerg and Finster, 1998), and mangroves (Kristensen et al., 1994), there are not many investigations looking directly at sulfate reduction associated with degrading macroalgae. Interestingly, a few studies have reported increased sulfate reduction associated with the decomposition of the green macroalga, *Ulva lactuca* (Nedergaard et al., 2002; Lomstein et al., 2006). Nedergaard et al. (2002) even found sulfate reduction to be more prominent with macroalgal-associated sulfate reducers than sediment associated and free living sulfate reducers during the degradation of *U. lactuca*, suggesting that the presence of sulfate reducers in macroalgal detrital systems may not be that uncommon. In this study, higher rates of BNF in the dark and possible microaerobic niches associated with *S. horneri* detritus suggested a potential role for sulfate reducers, which generally are thought to be active in anoxic zones (Canfield et al., 2010) but have also been observed in ostensibly oxic environments (Canfield and Marais, 1991).

Fulweiler et al. (2015) have demonstrated that the active microbial community can change by the end of an AR incubation and more specifically, the presence of C_2_H_2_, which is known to inhibit methanogens (Oremland and Capone, 1988), can result in up or down regulation of sulfur/sulfate reducers. However, despite potential changes induced by the presence C_2_H_2_, the use of sodium molybdate additions, a known inhibitor of sulfate reducers (Oremland and Capone, 1988), in the 2017 *S. horneri* decomposition experiment yielded reproducible instances of significantly reduced nitrogenase activity (∼53–98% inhibition). This suggests that sulfate reducers can contribute to BNF and at times, dominate the diazotrophic community in these detrital systems. Surprisingly, this translated into noticeable changes with the C:N ratio associated with *S. horneri* detritus, discussed below. Although sulfate reduction rates were not directly measured in this study, increasing inhibition of nitrogenase activity (up to 98% by day 12) throughout decomposition suggests that increasing sulfate reduction may also be associated with aging *S. horneri* detritus as observed by Nedergaard et al. (2002) and Lomstein et al. (2006) with *U. lactuca* decomposition. This suggests that sulfate reducers might be playing an important role in the breakdown of the more recalcitrant macroalgal detritus left toward the latter phases of macroalgal decomposition.

### Imprint of BNF on *S. horneri* Detritus

A common observation with aging macrophyte detritus is the decrease in C:N ratio throughout decomposition which has been linked, with varying extent, to the associated diazotrophic activity (Cundell et al., 1979; Kenworthy and Thayer, 1984; Kenworthy et al., 1987). Additionally, there have also been several studies that directly link BNF observed in these marine detrital systems to N immobilization of the aging detritus, highlighting the importance of diazotrophs in marine detrital food chains (Van der Valk and Attiwill, 1984; Woitchik et al., 1997; Pelegri and Twilley, 1998). Not surprisingly, this trend of decreasing C:N ratio (observed in this study with the 2017 *S. horneri* decomposition experiment, Figure 5C) has also been reported on several accounts with aging macroalgal detritus but not linked directly to BNF and instead, generally attributed to the buildup of microbial biomass (Smith and Foreman, 1984; Rieper-Kirchner, 1989; Duggins and Eckman, 1997; Banta et al., 2004). Alternatively, Rice (1982) argue that increases in detrital N cannot be fully accounted for by microbial protein but rather, depend more on the accumulation of non-labile N, which has also been suggested by others (Robinson et al., 1982; Smith and Foreman, 1984). In this study, changes in the %C, %N, C:N ratio, and δ^15^N alongside BNF rates from the second *S. horneri* decomposition experiment (Figures 4A,B, 5A–D) highlight the potential influence that diazotrophs may have on aging macroalgal detritus.

For instance, the largest decrease in C:N ratio, taking place over the first few days of decomposition, is accompanied by relatively little change in %C and is most likely driven by the steep increase in %N (Figures 5A–C). The BNF rates associated with dark and light control incubations on day 3 can account for ∼5 and 11%, respectively, of the observed N immobilization. On the contrary, parallel dark and light sodium molybdate incubations, where 66 and 88% of the nitrogenase activity was inhibited (Figures 4A,B), resulted in PID that were ∼24 and 28% more depleted in N, respectively, than the control treatments (Figures 5B,C). The sharpest decrease in δ^15^N of FD from 12.2‰ to 10.7‰ also occurred during this period (Figure 5D), further supporting that BNF may be playing a direct role in influencing the changes associated with *S. horneri* detrital N. On day 8, BNF can account for ∼2 and 1% of the observed increase in N with PID from dark and light control incubations. Interestingly, sodium molybdate amendments did not inhibit nitrogenase activity in the light (Figure 4B) and the corresponding BNF accounted for 4% of the N immobilization. These relatively low contributions suggest that BNF was not playing a significant role in N immobilization during this period.

Instead, potential increases in microbial biomass, characteristic of the second phase of decomposition, or retention of refractory N in aging *S. horneri* detritus might account for much of the N immobilization. Nonetheless, the lack of nitrogenase inhibition by sodium molybdate on day 8 resulted in a ∼11% more N enriched PID in the light than when BNF was strongly inhibited, 69%, in the dark (Figure 5B). Similarly, strong inhibition of nitrogenase activity on day 12, 95–98%, by sodium molybdate treatments (Figures 4A,B) resulted in PID that was ∼11–33% more N depleted (Figure 5B). This, alongside a smaller difference in C content between light and dark molybdate incubations, results in a significantly higher C:N ratio for PID when BNF is inhibited, suggesting that BNF could still indirectly be influencing the nutritional quality of the macroalgal detritus. While a few studies have suggested BNF as a potential source of N enrichment (Hill and McQuaid, 2009; Hamersley et al., 2015), our results provide direct evidence for how BNF, or therein lack of, can influence the nutritional quality of the aging *S. horneri* detritus.

## Conclusion

Investigating the role of diazotrophs with *S. horneri* throughout its life cycle has provided insight into the relevance of BNF with regard to the success of this globally relevant macroalga in a non-native ecosystem. Our findings suggest, at least during periods of N deficiency, that BNF can potentially provide significant amounts of fixed N to macroalgae and heterotrophic BNF associated with living macroalgae may be more significant than what is currently thought. Higher BNF rates observed with decomposing *Sargassum* in this study, which has also been reported in the past with pelagic *Sargassum* (Hanson, 1977; Phlips et al., 1986), suggests that DMAs with decomposing *Sargassum* species may be a global occurrence. Moreover, several studies reporting higher BNF activity associated with other senescent brown (Hamersley et al., 2015) and green (Zuberer and Silver, 1978) macroalgae further suggest that DMAs with aging macroalgal detritus may not be a rare phenomenon. Macroalgae such as *S. horneri* may provide a niche for BNF and these DMAs may ultimately allow for a more efficient decomposition of a large reservoir of organic material. Currently, the macroalgal detrital food web remains a vastly unexplored niche for N fixers. Our findings suggest that heterotrophic N fixers and sulfate reducing diazotrophs may be important members of macroalgal detrital diazotrophic communities. As most macroalgal production ultimately enters the detrital food web, future studies investigating DMAs with decomposing macroalgae are necessary to elucidate the role of BNF in these detrital systems.

## Author Contributions

YR, MM, and DC designed the research. YR conducted the research, and analyzed and synthesized the data. YR and DC wrote the manuscript.

## Conflict of Interest Statement

The authors declare that the research was conducted in the absence of any commercial or financial relationships that could be construed as a potential conflict of interest.
