# Supplementary material for: Diazotrophic Macroalgal Associations With Living and Decomposing Sargassum
Source: Front Microbiol. 2018 Dec 18;9:3127. doi: 10.3389/fmicb.2018.03127 (PMC6305716; doi:10.3389/fmicb.2018.03127)
Supplement: Supplementary file 1 [file Table_1.DOCX]

**Supplementary Table 1:** Details of sample collection.

| Date | Location | Latitude | Longitude | Species | Life Stage |
| --- | --- | --- | --- | --- | --- |
| 02/02/2016 | Isthmus Reef | 33.449 °N | 118.489 °W | *S. horneri* | Mature Adult |
| 03/25/2016 | Isthmus Reef | 33.449 °N | 118.489 °W | *S. horneri* | Mature Adult |
| 07/08/2016 | Rippers Cove | 33.428 °N | 118.433 °W | *S. horneri* | Senescent Adult |
| 07/08/2016 | Rippers Cove | 33.428 °N | 118.433 °W | *S. palmeri* | Senescent Adult |
| 06/18/2017 | Isthmus Reef | 33.449 °N | 118.489 °W | *S. horneri* | Senescent Adult |
| 07/27/2017 | Isthmus Reef | 33.449 °N | 118.489 °W | *S. horneri* | Juvenile (5 cm) |
| 08/02/2017 | Isthmus Reef | 33.449 °N | 118.489 °W | *S. horneri* | Juvenile (8 cm) |
| 09/13/2017 | Isthmus Reef | 33.449 °N | 118.489 °W | *S. horneri* | Juvenile (5-11 cm) |
| 09/13/2017 | Isthmus Reef | 33.449 °N | 118.489 °W | *S. horneri* | Immature |
| 10/18/2017 | Isthmus Reef | 33.449 °N | 118.489 °W | *S. horneri* | Juvenile (8-17 cm) |
| 10/18/2017 | Isthmus Reef | 33.449 °N | 118.489 °W | *S. horneri* | Immature |
| 11/30/2017 | Isthmus Reef | 33.449 °N | 118.489 °W | *S. horneri* | Juvenile (5-8 cm) |
| 11/30/2017 | Isthmus Reef | 33.449 °N | 118.489 °W | *S. horneri* | Immature |
| 12/8/2017 | Isthmus Reef | 33.449 °N | 118.489 °W | *S. horneri* | Juvenile (6 cm) |
