# Supplementary material for: Diazotrophic Macroalgal Associations With Living and Decomposing Sargassum
Source: Front Microbiol. 2018 Dec 18;9:3127. doi: 10.3389/fmicb.2018.03127 (PMC6305716; doi:10.3389/fmicb.2018.03127)
Supplement: Supplementary file 2 [file Table_2.docx]

**Supplementary Table 2:** BNF rates of control incubations (~48 hours) under dark (D) and light (L) treatments from different collections of *S. horneri* at various life stages. Rates are expressed as nmol N × g^-1^(dw) × h^-1^ ± SE. Not Detectable (ND).

| Day | Season | Life Stage | Light Treatment | Control |
| --- | --- | --- | --- | --- |
| 09/13/2017 | Fall | Juvenile (5-11 cm) | D & L | ND |
| 09/13/2017 | Fall | Immature | D & L | ND |
| 10/18/2017 | Fall | Juvenile (8-17 cm) | D & L | ND |
| 10/18/2017 | Fall | Immature | D & L | ND |
| 11/30/2017 | Fall | Juvenile (5-8 cm) | D & L | ND |
| 11/30/2017 | Fall | Immature | D & L | ND |
| 12/8/2017 | Winter | Juvenile (6 cm) | D & L | ND |
| 02/02/2016 | Winter | Mature Adult | D | 2.01 ± 0.99 |
| 02/02/2016 | Winter | Mature Adult | L | 0.73 ± 0.43 |
| 03/25/2016 | Spring | Mature Adult | D & L | ND |
| 06/18/2017 | Summer | Senescent Adult | D | 11.5 ± 8.27 |
| 06/18/2017 | Summer | Senescent Adult | L | 11.7 ± 8.64 |
| 07/08/2016 | Summer | Senescent Adult | D | 22.9 ± 10.2 |
| 07/08/2016 | Summer | Senescent Adult | L | 23.7 ± 5.04 |
| 07/27/2017 | Summer | Juvenile (5 cm) | D | 27.8 ± 4.12 |
| 07/27/2017 | Summer | Juvenile (5 cm) | L | 66.2 ± 11.7 |
| 08/02/2017 | Summer | Juvenile (8 cm) | D | 30.9 ± 3.28 |
| 08/02/2017 | Summer | Juvenile (8 cm) | L | 91.4 ± 21.1 |
