# Supplementary material for: Diazotrophic Macroalgal Associations With Living and Decomposing Sargassum
Source: Front Microbiol. 2018 Dec 18;9:3127. doi: 10.3389/fmicb.2018.03127 (PMC6305716; doi:10.3389/fmicb.2018.03127)
Supplement: Supplementary file 3 [file Table_3.docx]

**Supplementary Table 3:** BNF rates with glucose and mannitol amendments and their impact on BNF stimulation (Gx, Mx) under dark/light treatments for 2016 *S. horneri* & *S. palmeri* decomposition experiments. Rates are expressed as nmol N × g^-1^(dw) × h^-1^ ± SE. No Stimulation (NS), Not Detectable (ND) and Not Applicable (NA). Due to insufficient biomass, G/M amendments were not carried out on day 28 with *S. horneri* & *S. palmeri*.

| Day | Species | Light | Control | Glucose (G) | Mannitol (M) | Gx | Mx |
| --- | --- | --- | --- | --- | --- | --- | --- |
| 0 | *S. horneri* | Dark | 22.9 ± 10.2 | 66 ± 8.59 | 57 ± 8.3 | 3x | 2.5x |
| 5 | *S. horneri* | Dark | 77.8 ± 8.69 | 68.2 ± 3.19 | 41 ± 7.81 | NS | NS |
| 11 | *S. horneri* | Dark | 167 ± 20 | 113 ± 12.2 | 86.6 ± 13.8 | NS | NS |
| 21 | *S. horneri* | Dark | 1.26 ± 0.03 | 6.62 ± 2.77 | 6.89 ± 1.91 | 5.3x | 5.5x |
| 28 | *S. horneri* | Dark | ND | NA | NA | NA | NA |
| 0 | *S. horneri* | Light | 23.7 ± 5.04 | 19 ± 5.81 | 14.8 ± 11.4 | NS | NS |
| 5 | *S. horneri* | Light | 38.3 ± 8.24 | 68.2 ± 3.6 | 29.8 ± 0.09 | 1.8x | NS |
| 11 | *S. horneri* | Light | 60.9 ± 5.94 | 98.5 ± 19.2 | 98.8 ± 8.89 | 1.6x | 2x |
| 21 | *S. horneri* | Light | 2.08 ± 0.57 | 12.8 ± 4.11 | 31.7 ± 16.6 | 6.2x | 15x |
| 28 | *S. horneri* | Light | ND | NA | NA | NA | NA |
| 0 | *S. palmeri* | Dark | ND | ND | ND | NA | NA |
| 5 | *S. palmeri* | Dark | 17.2 ± 7.79 | 64 ± 10.9 | 50.8 ± 14 | 3.7x | 3x |
| 11 | *S. palmeri* | Dark | 35 ± 16.4 | 126 ± 20.8 | 107 ± 36 | 3.6x | 3.1x |
| 21 | *S. palmeri* | Dark | 3.69 ± 0.69 | 154 ± 39.8 | 139 ± 12.2 | 42x | 38x |
| 28 | *S. palmeri* | Dark | 2.3 ± 1.07 | NA | NA | NA | NA |
| 0 | *S. palmeri* | Light | 1.31 ± 1.07 | 1.77 ± 0.44 | 2.69 ± 2.12 | 1.4x | 2.1x |
| 5 | *S. palmeri* | Light | 44.9 ± 13.2 | 57.8 ± 12.7 | 47.9 ± 6.32 | 1.3x | NS |
| 11 | *S. palmeri* | Light | 65.3 ± 16.9 | 124 ± 55.6 | 109 ± 37.9 | 1.9x | 1.6x |
| 21 | *S. palmeri* | Light | 6.52 ± 0.98 | 237 ± 19.7 | 161 ± 2.12 | 36x | 25x |
| 28 | *S. palmeri* | Light | 8.32 ± 3.94 | NA | NA | NA | NA |
