# Supplementary material for: Diazotrophic Macroalgal Associations With Living and Decomposing Sargassum
Source: Front Microbiol. 2018 Dec 18;9:3127. doi: 10.3389/fmicb.2018.03127 (PMC6305716; doi:10.3389/fmicb.2018.03127)
Supplement: Supplementary file 4 [file Table_4.docx]

**Supplementary Table 4:** BNF rates for sodium molybdate additions and its inhibition on nitrogenase activity under dark/light treatment during the 2017 *S. horneri* decomposition experiment. Rates are expressed as nmol N × g^-1^(dw) × h^-1^ ± SE.

| Day | Light Treatment | Control | Molybdate | % Inhibition of BNF Rates |
| --- | --- | --- | --- | --- |
| 0 | Dark | 11.5 ± 8.27 | 5.39 ± 3.46 | 53 |
| 3 | Dark | 247 ± 27.2 | 83.8 ± 10.6 | 66 |
| 8 | Dark | 154 ± 9.78 | 47.6 ± 1.38 | 69 |
| 12 | Dark | 167 ± 34.2 | 3.44 ± 1.26 | 98 |
| 15 | Dark | 12.2 ± 4.91 | 17.1 ± 2.67 | No Inhibition |
| 0 | Light | 11.7 ± 8.64 | 89.7 ± 48.1 | No Inhibition |
| 3 | Light | 141 ± 59 | 17.5 ± 8.01 | 88 |
| 8 | Light | 80.4 ± 13.8 | 106 ± 10.4 | No Inhibition |
| 12 | Light | 171 ± 7.48 | 8.73 ± 1.83 | 95 |
| 15 | Light | 13.3 ± 4.67 | 13.6 ± 1.87 | No Inhibition |
