# Supplementary material for: Diazotrophic Macroalgal Associations With Living and Decomposing Sargassum
Source: Front Microbiol. 2018 Dec 18;9:3127. doi: 10.3389/fmicb.2018.03127 (PMC6305716; doi:10.3389/fmicb.2018.03127)
Supplement: Supplementary file 5 [file Table_5.DOCX]

**Supplementary Table 5:** Compilation of reported growth rates as g (dw) × day^-1^ (Umezaki 1984^1^, Yoshida, Yoshikawa, and Terawaki 2001^2a, b^) and estimations of growth rates as g (dw) × day^-1^ for samples collected for this study on 07/27/2017 * and 08/02/2017 ** using previously reported relative growth rate (RGR) expressed as % increase in fresh weight per day (Gao and Hua 1997^3^) or % increase in blade weights per day (Choi et al. 2008^4a, b^).

Daily biomass increase (DBI): whole sample dry weight (0.384 g *, 0.566 g **) × RGR (%) = g (dw) × day^-1^

| Duration | Location | DBI  g (dw) × day^-1^ | RGR (%) |
| --- | --- | --- | --- |
| ^1^ 08/03/1981-10/31/1981 | Obama Bay, Japan | 0.003 | NA |
| ^2a^ 08/10/1995-09/07/1995 | Hiroshima Bay, Japan | 0.01 | NA |
| ^2b^ 08/06/1996-09/09/1996 | Hiroshima Bay, Japan | 0.01 | NA |
| ^3^ 08/17/1987-08/25/1987 | Maizuru Bay, Japan | 0.0200 *  0.0294 ** | 5.2 |
| ^4a^ Laboratory | Jeonchonri, Wolsung, Kyungbuk,  Korea | 0.0154 *  0.0226** | 4 |
| ^4b^ Laboratory | Jeonchonri, Wolsung, Kyungbuk,  Korea | 0.0192 *  0.0283 ** | 5 |
