# Supplementary material for: Diazotrophic Macroalgal Associations With Living and Decomposing Sargassum
Source: Front Microbiol. 2018 Dec 18;9:3127. doi: 10.3389/fmicb.2018.03127 (PMC6305716; doi:10.3389/fmicb.2018.03127)
Supplement: Supplementary file 6 [file Table_6.docx]

**Supplementary Table 6:** Contributions of fixed N from diazotrophic activity to meet N demands for juvenile *S. horneri* collected on 07/27/2017 * and 08/02/2017 **.

N Requirement: average %N of *S. horneri* (1.014 ± 0.0381 % *, 1.066 ± 0.0691 % **) × growth rates listed in Supp. Table 5 as g (dw) × day^-1^ = g N × day^-1^

BNF Production ^A^: Average light and dark BNF rates (nmol N × g^-1^ (dw) × h^-1^) calculated using a 3 C_2_H_2_:1 N_2_ ratio (47.0 *, 61.1 **) × whole sample dry weight (0.384 g *, 0.566 g **) × molecular weight of N (g/nmol) × 24 hours = g of N fixed per day

BNF Production ^B^: Same calculation as BNF Production ^A^ except using BNF rates using a 4 C_2_H_2_: 1 N_2_ ratio yielding (nmol N × g^-1^ (dw) × h^-1^) of 35.3 * and 45.8 **.

% N Supplied by BNF ^A, B^: (BNF Production ^A, B^/N Requirement) × 100 = % contribution of N by BNF.

| Date of Collection | N Requirement  (g N × day^-1^) | BNF Production  (g N × day^-1^) ^A^ | BNF Production  (g N × day^-1^) ^B^ | % N Supplied by BNF ^A^ | % N Supplied by BNF ^B^ |
| --- | --- | --- | --- | --- | --- |
| 07/27/2017 | 3.041 E-05 ^1^ | 6.072 E-06 | 4.55 E-06 | 20.0 | 15.0 |
| 07/27/2017 | 1.014 E-04 ^2^ | 6.072 E-06 | 4.55 E-06 | 5.99 | 4.49 |
| 07/27/2017 | 2.025 E-04 ^3^ | 6.072 E-06 | 4.55 E-06 | 3.00 | 2.25 |
| 07/27/2017 | 1.56 E-04 ^4a^ | 6.072 E-06 | 4.55 E-06 | 3.90 | 2.92 |
| 07/27/2017 | 1.95 E-04 ^4b^ | 6.072 E-06 | 4.55 E-06 | 3.12 | 2.34 |
| 08/02/2017 | 3.20 E-05 ^1^ | 1.16 E-05 | 8.72 E-06 | 36.3 | 27.3 |
| 08/02/2017 | 1.066 E-04 ^2^ | 1.16 E-05 | 8.72 E-06 | 10.9 | 8.17 |
| 08/02/2017 | 3.14 E-04 ^3^ | 1.16 E-05 | 8.72 E-06 | 3.70 | 2.78 |
| 08/02/2017 | 2.41 E-04 ^4a^ | 1.16 E-05 | 8.72 E-06 | 4.82 | 3.61 |
| 08/02/2017 | 3.017 E-04 ^4b^ | 1.16 E-05 | 8.72 E-06 | 3.85 | 2.89 |
| Average | 1.68 E-04 | 8.85 E-06 | 6.64 E-06 | 9.56 | 7.17 |
