# Supplementary material for: Diazotrophic Macroalgal Associations With Living and Decomposing Sargassum
Source: Front Microbiol. 2018 Dec 18;9:3127. doi: 10.3389/fmicb.2018.03127 (PMC6305716; doi:10.3389/fmicb.2018.03127)
Supplement: Supplementary file 7 [file Table_7.DOCX]

**Supplementary Table 7:** %C, %N, C:N ratio and δ^15^N values for samples (FD) before the start of dark (D) and light (L) incubations for ~48 hours under control (Con) and sodium molybdate (Mol) treatments and samples at the end of the incubation (PID) throughout the 2017 *S. horneri* decomposition experiment.

| Day | Sample | Light | Treatment | %C | %N | C:N Ratio | δ^15^N |
| --- | --- | --- | --- | --- | --- | --- | --- |
| 0 | FD | NA | NA | 29.0 ± 0.1 | 1.45 ± 0.02 | 19.9 ± 0.25 | 12.2 ± 0.87 |
| 3 | FD | NA | NA | 28.5 ± 0.06 | 1.72 ± 0.03 | 16.6 ± 0.32 | 10.7 ± 0.11 |
| 8 | FD | NA | NA | 28.5 ± 0.2 | 1.75 ± 0.03 | 16.3 ± 0.34 | 10.6 ± 0.79 |
| 12 | FD | NA | NA | 28.2 ± 0.13 | 2.05 ± 0.01 | 13.8 ± 0.05 | 9.83 ± 0.19 |
| 15 | FD | NA | NA | 33.8 ± 0.16 | 2.33 ± 0.03 | 14.5 ± 0.15 | 9.25 ± 0.23 |
| 2 | PID | D | Con | 25.8 ± 0.44 | 1.29 ± 0.02 | 20.0 ± 0.53 | NA |
| 5 | PID | D | Con | 25.8 ± 0.23 | 2.02 ± 0.02 | 12.8 ± 0.06 | NA |
| 10 | PID | D | Con | 25.0 ± 0.92 | 2.25 ± 0.09 | 11.1 ± 0.07 | NA |
| 14 | PID | D | Con | 21.5 ± 0.74 | 1.70 ± 0.09 | 12.7 ± 0.48 | NA |
| 17 | PID | D | Con | 23.7 ± 0.42 | 1.74 ± 0.05 | 13.6 ± 0.20 | NA |
| 2 | PID | D | Mol | 23.7 ± 0.92 | 1.13 ± 0.06 | 21.1 ± 0.61 | NA |
| 5 | PID | D | Mol | 23.2 ± 0.08 | 1.30 ± 0.10 | 18.1 ± 1.47 | NA |
| 10 | PID | D | Mol | 23.7 ± 0.87 | 1.71 ± 0.09 | 13.9 ± 0.27 | NA |
| 14 | PID | D | Mol | 22.3 ± 0.32 | 1.51 ± 0.06 | 14.8 ± 0.41 | NA |
| 17 | PID | D | Mol | 23.9 ± 0.89 | 1.57 ± 0.06 | 15.3 ± 1.08 | NA |
| 2 | PID | L | Con | 27.9 ± 0.79 | 1.38 ± 0.02 | 20.1 ± 0.48 | NA |
| 5 | PID | L | Con | 26.9 ± 0.97 | 1.8 ± 0.06 | 15.0 ± 0.21 | NA |
| 10 | PID | L | Con | 26.1 ± 1.32 | 2.32 ± 0.17 | 11.4 ± 0.41 | NA |
| 14 | PID | L | Con | 24.3 ± 0.8 | 2.10 ± 0.07 | 11.8 ± 0.18 | NA |
| 17 | PID | L | Con | 25.2 ± 0.61 | 1.64 ± 0.04 | 15.4 ± 0.23 | NA |
| 2 | PID | L | Mol | 23.4 ± 0.29 | 1.19 ± 0.06 | 19.7 ± 1.03 | NA |
| 5 | PID | L | Mol | 22.2 ± 0.41 | 1.25 ± 0.14 | 18.3 ± 2.42 | NA |
| 10 | PID | L | Mol | 23.1 ± 0.75 | 1.93 ± 0.15 | 12.1 ± 0.53 | NA |
| 14 | PID | L | Mol | 21.2 ± 1.19 | 1.38 ± 0.08 | 15.3 ± 0.50 | NA |
| 17 | PID | L | Mol | 23.0 ± 0.92 | 1.46 ± 0.08 | 15.9 ± 1.14 | NA |
