# Supplementary material for: Diazotrophic Macroalgal Associations With Living and Decomposing Sargassum
Source: Front Microbiol. 2018 Dec 18;9:3127. doi: 10.3389/fmicb.2018.03127 (PMC6305716; doi:10.3389/fmicb.2018.03127)
Supplement: Supplementary file 8 [file Table_8.docx]

**Supplementary Table 8:** Theoretical percent contribution to N immobilization (Imm.) by BNF in the various treatments during the 2017 *S. horneri* decomposition experiment: dark/light control (DC/LC) and dark/light molybdate (DM/LM).

All calculations are based on 1 g (dry weight, dw) of *S. horneri* detritus

Theoretical mg of N from BNF: (Average BNF rates corresponding to the various treatments, from Supp. Table 4) × 1 g (dw) × 48 hours × (14.001 g of N/1,000,000,000 nmol of N) × (1,000 mg/1g)

Δ mg N: ((mean %N of PID/100) × 1 g (dw) × (1,000 mg/1g)) - ((mean %N of FD/100) × 1 g (dw) × (1,000 mg/1g)) + Theoretical mg of N from BNF = Δ mg N for the various treatments

% N Imm.: Theoretical mg of N from BNF × (1/ Δ mg N) × 100 = % N Imm. for the various treatments

| Day | Treatment | FD N (mg) | PID N (mg) | N (mg) from BNF | Δ mg N | % N Imm. |
| --- | --- | --- | --- | --- | --- | --- |
| 3 | DC | 17.2 | 20.2 | 0.166 | 3.17 | 5.24 |
| 3 | LC | 17.2 | 18.0 | 0.095 | 0.895 | 10.6 |
| 8 | DC | 17.5 | 22.5 | 0.104 | 5.10 | 2.04 |
| 8 | LC | 17.5 | 23.2 | 0.054 | 5.75 | 0.938 |
| 8 | LM | 17.5 | 19.3 | 0.071 | 1.87 | 3.80 |
