# Supplementary figures and images for: Diazotrophic Macroalgal Associations With Living and Decomposing Sargassum
Source: Front Microbiol. 2018 Dec 18;9:3127. doi: 10.3389/fmicb.2018.03127 (PMC6305716; doi:10.3389/fmicb.2018.03127)

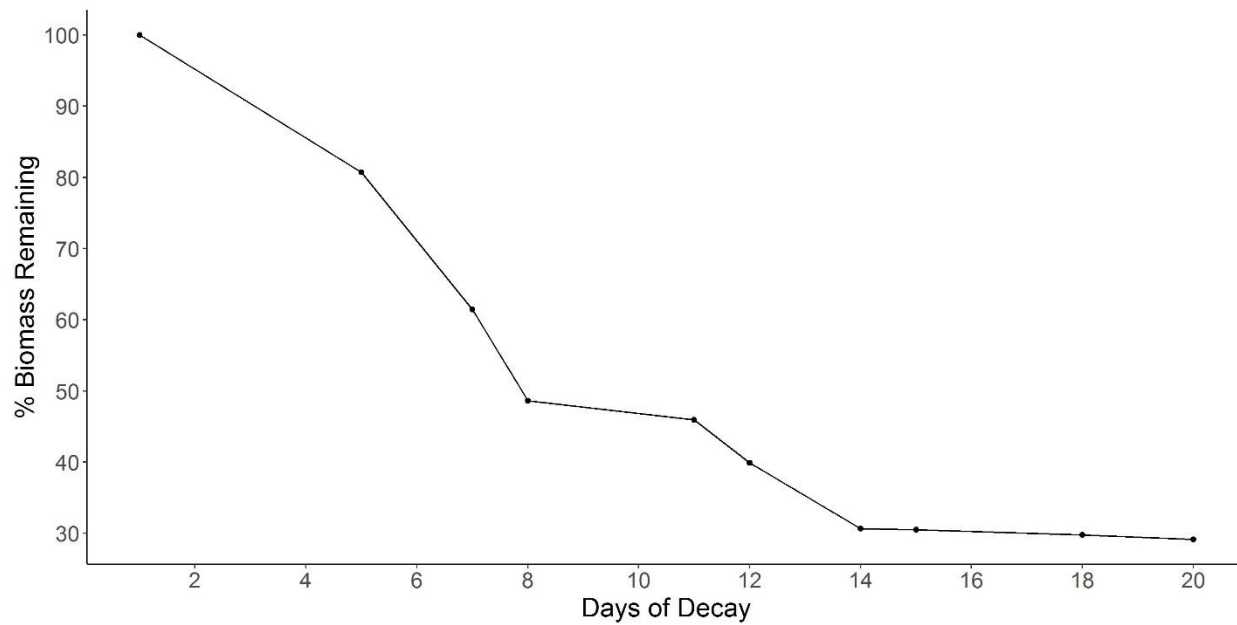

*Supplementary Figure 1: Percent loss in biomass of S. horneri throughout decomposition.*

Supplement: Supplementary file 9 [file Data_Sheet_1.PDF]
