# Supplementary material for: Diazotrophic Macroalgal Associations With Living and Decomposing Sargassum
Source: Front Microbiol. 2018 Dec 18;9:3127. doi: 10.3389/fmicb.2018.03127 (PMC6305716; doi:10.3389/fmicb.2018.03127)
Supplement: Supplementary file 10 [file Data_Sheet_2.PDF]

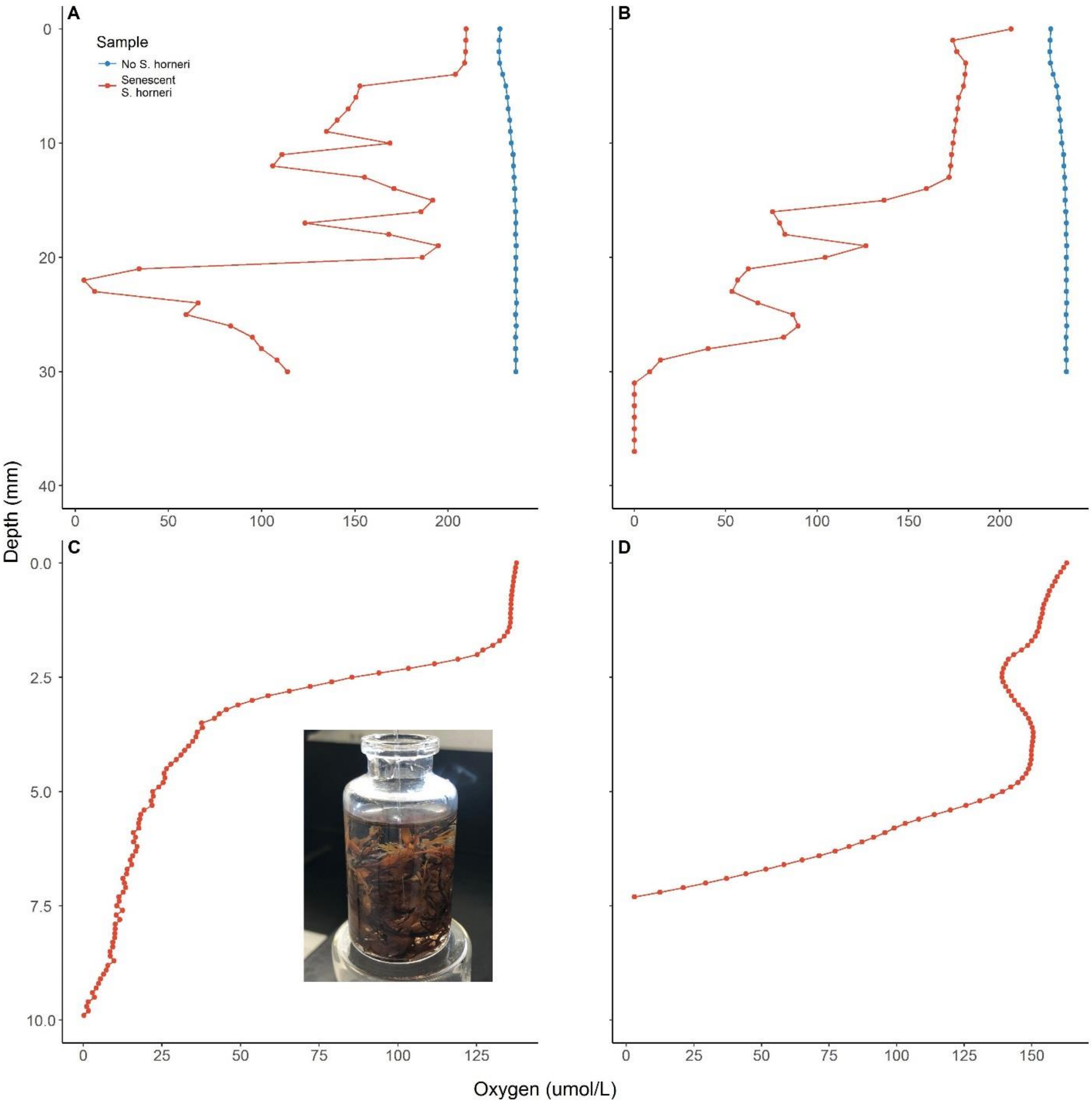

Supplementary Figure 2: Variations in oxygen (O_2) concentrations surrounding senescent S. horneri *in an open serum vial in the light (A&B, blue profile representing O_2 concentrations of just the seawater without any senescent seaweed present). Finer (µm) resolution snapshots of O_2 concentrations approaching senescent* S. horneri *detritus (C&D).*
